# Association between socioeconomic status and diet quality in Mexican men and women: A cross-sectional study

**DOI:** 10.1371/journal.pone.0224385

**Published:** 2019-10-23

**Authors:** Nancy López-Olmedo, Barry M. Popkin, Lindsey Smith Taillie

**Affiliations:** Department of Nutrition, University of North Carolina at Chapel Hill, Chapel Hill, North Carolina, United Stated of America; National Institute of Health and Nutrition, National Institutes of Biomedical Innovation, Health and Nutrition, JAPAN

## Abstract

Examining the potential differences in diet quality among socioeconomic status (SES) subgroups in Mexican adults may help to explain SES disparities in the burden of non-communicable diseases. We determined the association between SES, gender and diet quality among Mexican adults. We analyzed data from adults participating in the subsample with dietary information from the Mexican National Health and Nutrition Survey 2012 (n = 2,400), and developed the Mexican Diet Quality Index based on the Mexican Dietary Guidelines. We tested the interaction between sex and SES indicators using multivariable linear regression models. Sex was not a modifier; therefore, the analyses were carried out in the overall sample of men and women. The mean age was 42 (SE = 0.4) years, the total diet quality score was 38 (SE = 0.4), and a high percentage of men and women were classified with reading/writing skills or 3–9 years of school. A higher percentage of adults were classified with high versus medium or low assets index. In the multivariable model further adjusted for the assets index, for adults with education in the reading and/or 3–9 years of schooling and those with ≥10 years of school, there was a 3.7 and 5.8 points lower total diet quality score than with adults with no reading/writing skills (*p* < 0.05). Likewise, in multivariable model further adjusted for educational level, the total diet quality score was 2.5 points and 3.3 points lower in adults classified with medium and high assets index, respectively, versus low assets index (*p* < 0.05). The difference between individuals with medium and high assets index was not statistically significant. Although there is currently better diet quality among adults with low SES, this needs to continue to be monitored as Mexico progress through the nutrition transition.

## Introduction

Globally, in 2017, poor diets (diets high in saturated fats, added sugars, or sodium, but low in fruits, vegetables, legumes, or whole grains) were responsible for 22% of all deaths and 15% of all disability-adjusted life-years (DALYS) among adults [1]. In Mexico, a middle-income country, poor diets were the third leading risk factor for DALYS among adults (10.6%), after high body mass index (11.7%) and high fasting glucose (11.1%), both of which are also partially linked with dietary patterns [2]. In addition, a transition of the non-communicable disease (NCD) burden from high to low socioeconomic groups over time has been documented in Mexico and many other high and middle-income countries [3, 4]. Therefore, improving diets is pivotal to prevent or ameliorate adverse health outcomes, especially in low socioeconomic status (SES) groups which have much more untreated or poorly treated NCDs [5, 6].

The intakes of sugar-sweetened beverages and energy-dense nutrient-poor foods are especially problematic and have been widely studied in Mexico. National surveys indicate that the intake of caloric beverages increased 38% (from 250 kcal to 337 kcal per capita per day) from 1999 to 2012 [7]. Furthermore, even though the corn tortilla was still an important staple among Mexican adults in 2012 (representing 20% of total energy intake), the contribution of foods high in saturated fat or added sugars to the total energy intake was also high (16.1%) in all age groups [8]. However, nutrients or foods are not eaten in isolation and their intake may have synergistic or antagonistic effects [9, 10]. For this reason, the major focus of extensive research in recent years has been to measure the overall quality of the diet in a comprehensive manner by using dietary indices or scores developed to reflect the relation between a variety of foods and nutrients and health outcomes [11, 12]. These dietary indices have been widely used for predicting NCDs, including cardiovascular disease and type 2 diabetes [13–15]. Despite the usefulness of the dietary indices, the last time they were used to analyze the diet in a representative sample of Mexican adults was with data collected in 2006 [16].

A few studies have explored the potential differences in diet quality among socioeconomic subgroups in Mexican adults, which may help to explain disparities in the burden of NCDs [17]. For example, studies from high-income countries have found that higher SES was associated with higher diet quality [18–21]. In contrast, in middle-income countries, the results have been inconsistent [22, 23]. Moreover, educational level has been barely used to examine Mexicans, even though education may facilitate the acquisition of positive psycho-social and economic skills as well as healthier lifestyles, including better diet quality [24–26]. It is important to use multiple indicators of SES in order to be able to more comprehensively characterize the relationship between SES and dietary quality, and in particular in order to be able to tailor potential interventions to reduce diet-related healthy disparities among low-SES subpopulations.

It is also important to consider the potential sex-differences on diet quality for the formulation of suitable interventional programs among adults. Sex differences might be not only biological, but also gender-related. For instance, women may have a better diet quality than men given that women may be more concerned with the quality of their food [27]. What has been less studied is the potential modifying effect of sex on the association between SES and diet quality. Despite changes on gender roles over the last decades, men and women within the same SES might still have differential access to material, social, and psychological resources, as well as different distributions of health-related behaviors [28]. Based on previous research, we hypothesized that the relationship between SES and diet quality would be different for men and women. Although for men, there is no previous research on the link between SES and diet quality, one study found that socioeconomic indicators were positively associated with body mass index among Mexican men, whereas mixed results have been found in women [29]. Given that diet can be a mediator between SES and obesity, and lower diet quality can be associated with higher prevalence of obesity, we expected to observe an inverse association between SES and diet quality for men. For women, previous studies have not found significant linear association between SES and diet quality score [22, 30]. Specifically, it has been observed that diet quality is lower among women in the extreme categories of education [30]. On one hand, women with low educational level may be more likely to keep the traditional gender role of the household responsibilities and income constraints that can result in lower diet quality [30, 31]. On the other hand, women with high educational level may be more likely to have paid work but also household responsibilities, which could limit their time to cook or buy healthier foods and therefore lead to lower diet quality. Thus, it will be important to understand whether the relationship between SES and diet quality varies by sex in the Mexican population.

In this paper, using data from the 2012 Mexico National Health and Nutrition Survey, the last survey with dietary information publicly available, we examined the cross-sectional association between SES and diet quality among Mexican men and women. This study used educational level and assets index as indicators of SES, and the Mexican Diet Quality Index, which is based on the intake of foods relative to the current dietary guidelines for Mexico, to examine the diet quality in Mexican adults. We hypothesized that the educational level and the assets index would be positively associated with diet quality. Furthermore, we hypothesized that higher SES would be associated with lower total diet quality among men. In contrast, we hypothesized for women that increases in SES would be linked with improved total diet quality because of the way SES is more strongly linked with health awareness in women compared with men [32].

## Methods

### Study design and population

The National Health and Nutrition Survey (ENSANUT, by its Spanish acronym Encuesta Nacional de Salud y Nutrición) 2012 contains information about sociodemographic characteristics, nutrition, and health from 96,031 people randomly selected (46,303 adults), and dietary information was obtained from a random subsample (10,886 individuals, 3,174 adults) representative of the national, regional, and urban/rural population [33]. Data are available under request on the ENSANUT website (https://ensanut.insp.mx/basesdoctos.php). All verbal responses by participants were collected by trained interviewers. Written informed consent was obtained for each eligible person 18 years and older, and the survey protocol was approved by the Ethics Committee of the National Institute of Public Health in Mexico.

We included non-pregnant and non-lactating adults aged twenty to sixty-nine years with information on educational level, literacy, and diet (*n* = 2,676). We excluded individuals who were missing data on smoking status (*n* = 154) or body mass index (*n* = 95). We also excluded those with a ratio of total energy intake to estimated energy requirement (in logarithmic scale) below -3 SD and above +3 SD (*n* = 30), as previously described [34]. Some subjects were excluded for two or more variables; therefore, the total number of excluded participants does not equal the sum of all the excluded participants by variable. The analytic sample was composed of 2,400 adults.

### Study variables

#### Diet quality score

Dietary information was collected using the twenty-four-hour dietary recall developed by the USDA (automated 5-step multiple-pass method), adapted to the Mexican context [35, 36]. Interviewers also collected a second twenty-four-hour dietary recall in a random subsample of ~ 9% of participants with the first twenty-four-hour dietary recall. For this study, we used information from the first twenty-four-hour recall only. Persons 15 years of age and older were asked about their intake, while persons in charge of food preparation and distribution in the household were asked about ingredients and recipes of foods prepared at home [34]. Energy and nutrient intake were calculated using the food composition database compiled by the National Institute of Public Health from different sources [37–40].

We developed the Mexican Diet Quality Index based on the Mexican Dietary Guidelines, which were published in 2015 [41]. These guidelines contain the number and size of servings recommended for nine food groups, by age group and total energy intake. The food groups include fruit, vegetables, cereals, legumes, food of animal origin (i.e., meat products), dairy, tap water, sugars, and fats. We did not consider tap water as a Mexican Diet Quality Index component because the Mexican Dietary Guidelines only include a suggested range of water consumption (750–2,000 mL), since water needs can vary by age, physical activity, and weather. Furthermore, we used the number of servings recommended for adults with a total energy intake requirement of 2,000 kcal per day (See S1 Table for more details) to create thirteen diet quality components. We developed two fruit-related Mexican Diet Quality Index components: whole fruit and 100% fruit juice, considering that the latter is recommended to be consumed in moderation (<125 mL/day). We determined the criterion for 100% fruit juices minimum score based on the recommendation of a panel of experts, who advised to limit their consumption to no more than 8 fl oz (~ 237 mL) [42]. Furthermore, we created two Mexican Diet Quality Index components for cereals, whole-grain and refined-grain, since the Mexican Dietary Guidelines indicate that at least half of cereals consumed should be whole grain. The committee expert, which developed the Mexican Dietary Guidelines, also recommends the consumption of meat products with lower saturated fat content, including seafood, poultry, and eggs. Less than half of meat products should be with high saturated or sodium content. Accordingly, we created two groups of meat products: seafood, poultry and eggs, and red and processed meat. The maximum of servings recommended for sugars is based on the latest recommendations of added sugars intake by WHO [43]. Therefore, we specified the group of “sugars” as “added sugars”, and the criteria for minimum and maximum scores were defined according to the percentage of their consumption from the total energy intake instead of the number of servings. Likewise, we used cut-off points recommended by WHO, as well as recommendations of fat intake for Mexican population to define minimum and maximum scores for polyunsaturated fat, saturated fat, and added sugars [43–45]. Finally, we created a diet quality component for sodium intake since the Mexican Dietary Guidelines, in line with international guidelines, recommend consuming no more than 2,000 mg of sodium. We defined the minimum score for sodium based on the results of systematic reviews and meta-analyses [46]. We defined scores between 0 (non-compliance) and 15 (intakes close to recommended) to each component. Specifically, we assigned a maximum score of five to those Mexican Diet Quality Index components derived from the same food group (e.g., whole-grain and refined-grain cereals). Furthermore, added sugars and sodium components were assigned with a maximum score of fifteen, given their high consumption among Mexican population [47, 48], and therefore their potential impact on health. (Table 1).

**Table 1:** Mexican Diet Quality Index.

Food component	Maximum points	Criteria for minimum score (0)	Criteria for maximum score
***Adequacy***			
Vegetables	10	0 servings	≥ 3 servings per 2,000 kcal
Whole fruit	10	0 servings	≥ 3 servings per 2,000 kcal
Whole-grain cereals	5	0 servings	≥ 3 servings per 2,000 kcal
Legumes	10	0 servings	≥ 2 servings per 2,000 kcal
Seafood, poultry or eggs	5	< 1 serving per 2,000 kcal	≥ 2 servings per 2,000 kcal
Low-fat dairy	5	0 servings	≥ 3.5 servings per 2,000 kcal
Polyunsaturated fat^a^	5	< 6% of total energy intake	> 10% of total energy intake
***Moderation***			
100% fruit juices	5	> 250 mL per 2,000 kcal	≤ 125 mL per 2,000 kcal
Sugar-sweetened beverages			
Refined grains	5	> 3 servings per 2,000 kcal	≤ 1 serving per 2,000 kcal
Red and processed meat	5	> 1.5 servings per 2,000 kcal	≤ 0.5 serving per 2,000 kcal
Added sugars	15	> 10% of total energy intake	< 5% of total energy intake
Sodium	15	> 2 g per 2,000 kcal	≤ 1.5 g per 2,000 kcal
Saturated fat	5	> 10% of total energy intake	< 7% of total energy intake
Total	100	0	100

#### Educational level

Educational attainment was ascertained by asking the question “What was the last year and grade you passed at school?”. ENSANUT also includes a question about literacy (“Do you know how to read and write?”). We defined educational level based on these two questions into the following categories: no reading/writing skills, reading/writing skills or 3–9 years of school (elementary and middle school that represent basic education in Mexico) and ≥ 10 years of school (high school or more). We selected these categories based on studies about the social and economic impact of illiteracy in Latin American and Caribbean region [49, 50]. Specifically, we considered in the category of “no reading/writing skills” not only to adults who answered that did not know how to read and write but also to those individuals with less than 2 years of elementary school that could have learned how to read and write, but that could have limited skills to understand texts with a certain degree of difficulty [49]. From this perspective, literacy is not only related to know how to read and write, but to acquire skills for effective social and productive performance of people in society [49].

#### Assets index

An assets index was constructed using factor analysis, where factor scores were estimated using a principal components approach. This methodology has been validated and previously described to define the SES in Mexican population [51]. Briefly, the assets index includes variables related to housing conditions (such as flooring and roofing materials), ownership of home appliances (refrigerator, stove, washing machine, television, radio, video player, telephone, and computer) and the number of rooms (other than bathroom, kitchen, and corridors). The index score was computed for each respondent and respondents were then classified into three categories (low, medium, and high) using tertiles of the distribution of the assets index scores as cut-off points.

### Covariates

The ENSANUT 2012 includes diverse information about sociodemographic factors through questionnaires. For our analyses, we considered age, sex, employment status, marital status, smoking status, body mass index (BMI), alcohol intake, region and area of residence, physical activity, and time spent sitting. The best functional form to use age (continuous, square, or categorical) was selected based on the Akaike Information Criterion. We categorized employment status as employed, homemaker, or other (retired, student, seeking a job), marital status as married, in union, separated / divorced /widowed, or single, and smoking status as current, former, or never smoker. BMI was categorized based on WHO definitions (normal, overweight, or obesity) [52], and alcohol intake was considered a dichotomous variable (whether or not the participants reported alcohol intake in the twenty-four-hour dietary recall). Locations with < 2,500 inhabitants were classified as rural and locations with ≥ 2,500 inhabitants as urban, and we defined regions as North, Central, and South. Finally, physical activity and time spent sitting (as a proxy of sedentary behavior) were assessed using the Spanish short version of the International Physical Activity Questionnaire [53]. Physical activity was classified based on WHO recommendations (inactive, moderately active, and active) [54], whereas time spent sitting was categorized based on Medina et al. approach [55]. Time spent sitting per day was divided into deciles and respondents in the highest decile (≥ 525 minutes per day, 8.75 hours per day) were categorized as having high sitting time; others were categorized as having low sitting time.

### Statistical analysis

We conducted all the analyses in Stata 15.0 (StataCorp, Stata Statistical Software, Release 15, 2017). We used population-weighted factors and considered the survey’s complex sampling design for all statistical analyses to generate nationally representative results. First, we analyzed the potential modifier effect of sex on the association of educational level and assets index with diet quality scores. We did not find that sex modified the association between socioeconomic indicators and diet quality; therefore, the successive analyses were carried out in the overall sample of men and women. We descriptively examined the total diet quality score, sociodemographic variables, and lifestyle behaviors. In order to determine the association of educational level and tertiles of assets index with overall diet quality scores, we performed the following linear regression models: 1) unadjusted (models 1), 2) adjusted for age (continuous) and sex, total energy intake, alcohol intake categories, employment status, marital status, smoking status, area and region of residence (models 2, multivariable-adjusted), and 3) models where the association between educational level and diet quality was further adjusted for tertiles of assets index (multivariable-adjusted + assets index) and models where the association between assets index and diet quality was further adjusted for educational level categories (multivariable-adjusted + educational level), to estimate how much each one of these two indicators of SES would explain the variability in diet quality scores. To explore which dietary components might explain more the variability of the overall diet quality score, we analyzed the association of the SES indicators with each one of the thirteen dietary components in multivariable-adjusted + assets index or educational level models. Adjusted diet quality means were calculated using the margins command in Stata. Furthermore, we used *t* tests, included in linear regression models, to compare dietary scores across educational levels and tertiles of assets index. Statistical tests were two-tailed and considered significant at *p* < 0.05, Bonferroni-adjusted for multiple comparisons.

### Sensitivity analyses

We conducted analyses in which corn tortilla was treated as a refined grain instead of a whole-grain cereal since it is uncertain whether all corn tortillas are made with whole grains. In addition, we conducted analyses to test whether the inclusion of physical activity and time spent sitting in models altered the association of educational levels and tertiles of assets index with total diet quality score. We did not include physical activity and time spent sitting in the main analyses due to the poor validity of the International Physical Activity Questionnaire among Mexican adults [56]. However, physical activity and time spent sitting could be important confounders of the relationship between education and diet quality. Neither did we consider the BMI categories in the main analyses because we considered the diet quality as a risk factor for obesity. However, given the cross-sectional design of our study, we further adjusted the models for BMI to test whether the inclusion of this covariate altered the associations between socioeconomic indicators and diet quality. Finally, we further analyzed the association between educational level and diet quality by separating the category of “reading/writing skills or 3–9 years of school” into “reading/writing skills or 3–6 years of school” and “7–9 years of school”, given that the role of diet behaviors could be different depending on whether the highest level of education was elementary or middle school.

## Results

The characteristics of the Mexican adults are displayed in Table 2. The mean of total diet quality score was 38±0.4, and a higher percentage of adults were classified with reading/writing skills or 3–9 years of school. Moreover, a higher percentage of adults were classified with high assets index. The Mexican adults studied as part of the ENSANUT 2012 consisted of a higher proportion of individuals living in the Central region than in the North and South, and living in urban than rural areas.

**Table 2:** Characteristics of Mexican adults (*n* = 2,400).

Age (years), mean (SE)	42 (0.4)
Men, % (SE)	45 (1.6)
Total diet quality score, mean (SE)	38 (0.4)
Educational level, % (SE)	
No reading / writing skills	8 (0.7)
Reading / writing skills or 3-9y of school	63 (1.5)
≥10 y of school	29 (1.4)
Tertiles of assets index, % (SE)	
Low	30 (1.2)
Medium	30 (1.3)
High	40 (1.6)
Employment status, % (SE)	
Employed	54 (1.5)
Homemaker	34 (1.4)
Other	12 (1.1)
Marital status, % (SE)	
Married	51 (1.5)
In union	19 (1.2)
Separated / divorced / widowed	11 (1.0)
Single	19 (1.3)
Smoking status, % (SE)	
Never	67 (1.5)
Former	18 (1.2)
Current	15 (1.2)
BMI categories, % (SE)	
Normal	29 (1.4)
Overweight	39 (1.5)
Obesity	32 (1.4)
Area, % (SE)	
Urban	74 (0.8)
Rural	26 (0.8)
Region, % (SE)	
North	20 (0.7)
Central	48 (1.0)
South	33 (0.9)
Physical activity, % (SE)	*n* = 2,221
Inactive	14 (1.1)
Moderately active	11 (1.0)
Active	75 (1.4)
Time spent sitting categories, % (SE)	
High sitting time	89 (1.0)
Low sitting time	11 (1.0

The association between educational level and total diet quality score is presented in Table 3. Educational level was inversely associated with total diet quality score in unadjusted and multivariable-adjusted models. In multi-variable adjustment model, the total diet quality score was, on average, 4.2 and 7 points lower in adults with reading/writing skills or 3–9 years of school and with ≥ 10 years of school, respectively, in comparison with adults with no reading/writing skills (*p* < 0.05). Likewise, the diet quality score was 2.1 points lower among individuals with ≥ 10 years of school in comparison with adults with reading/writing skills or 3–9 years of school (*p* < 0.05). After further adjusting for tertiles of assets index, the total diet quality score was 3.7 and 5.8 points lower in adults with reading/writing skills or 3–9 years of school and with ≥ 10 years of school, respectively, in comparison with adults with no reading/writing skills (*p* < 0.05). However, the diet quality score was not statistically different between adults with reading/writing skills or 3–9 years of school and with ≥ 10 years of school.

**Table 3:** Total diet quality score by educational level in Mexican adults (*n* = 2,400)^1^.

	Educational level with literacy		
	No reading/writing skills	Reading/writing skills or 3–9 y of school	≥ 10 y of school
	Mean (95% CI)	Mean (95% CI)	Mean (95% CI)
Model 1 (undajusted)	46.1 (43.4, 48.7)^a^	38.6 (37.6, 39.6)^b^	33.6 (32.2, 35.1)^c^
Model 2 (multivariable-adjusted)^2^	42.4 (39.8, 45.0)^a^	38.2 (37.2, 39.2)^b^	35.4 (33.9, 36.9)^c^
Model 3 (model 2 + tertiles of assets index)	41.7 (39.0, 44.3)^a^	38.0 (37.1, 39.0)^b^	35.9 (34.4, 37.5)^b^

^1^ Linear regression models were used to predict the mean diet quality score according to educational level with literacy categories. Weights were used to generate nationally representative results. Labeled means in a row without a common superscript letter (a,b,c) differ between educational levels, *p* < 0.05, Bonferroni adjusted.

^2^ Adjusted for age (continuous), sex, total energy intake, alcohol intake (yes, no), smoking status (current, former, never), employment status (employed, homemaker, other), marital status (married, in union, separated/divorced/widowed, single), region of residence (North, Central, South), area of residence (rural/urban).

Results of the association between tertiles of assets index and total diet quality score are shown in Table 4. A higher assets index was associated with lower total diet quality score. In the unadjusted model, the mean total diet quality score was, on average, 5.4 points and 8.3 points lower in adults classified with medium and high assets index, respectively, versus low assets index (*p* < 0.05). Likewise, the diet quality score was 2.9 lower in adults with high versus medium assets index (*p* < 0.05). In the multivariable-adjusted model, the mean total diet quality score was 2.9 points and 4.5 points lower in adults classified with medium and high assets index, respectively, versus low assets index (*p* < 0.05). The difference between individuals with medium and high assets index was no longer statistically significant. Similar results to those observed in the multivariable-adjusted model were observed when the model was further adjusted for educational level.

**Table 4:** Total diet quality score by tertiles of assets index in Mexican adults (*n* = 2,400)^1^.

	Educational level with literacy		
	Low	Medium	High
	Mean (95% CI)	Mean (95% CI)	Mean (95% CI)
Model 1 (undajusted)	42.7 (41.3, 44.1)^a^	37.3 (35.9, 38.7)^b^	34.4 (33.1, 35.7)^c^
Model 2 (multivariable-adjusted)^2^	40.4 (36.2, 38.8)^a^	37.5 (36.2, 38.8)^b^	35.9 (34.7, 37.2)^b^
Model 3 (model 2 + tertiles of assets index)	39.8 (38.3, 41.3)^a^	37.3 (36.0, 38.7)^b^	36.5 (35.2, 37.8)^b^

^1^ Linear regression models were used to predict the mean diet quality score according to assets index categories. Weights were used to generate nationally representative results. Labeled means in a row without a common superscript letter (a,b,c) differ between educational levels, *p* < 0.05, Bonferroni adjusted.

^2^ Adjusted for age (continuous), sex, total energy intake, alcohol intake (yes, no), smoking status (current, former, never), employment status (employed, homemaker, other), marital status (married, in union, separated/divorced/widowed, single), region of residence (North, Central, South), area of residence (rural/urban).

**Fig 1:**
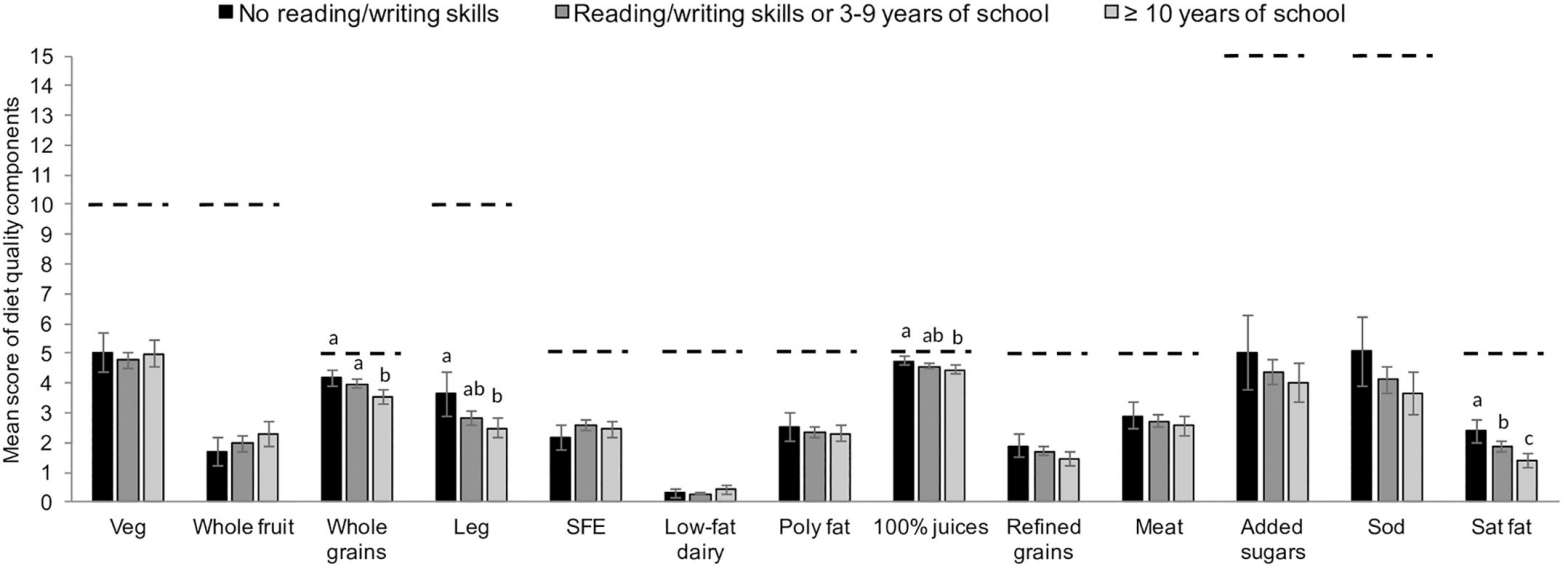
Predicted mean score of diet quality components by educational level. Predicted means ± 95% confidence intervals of diet quality components by educational level among adults in multivariable-adjusted + tertiles of assets index models. Predicted values were obtained using linear regression models adjusted for age (continuous), sex, total energy intake, alcohol intake (yes, no), smoking status (current, former, never), employment status (employed, homemaker, other), marital status (married, in union, separated/divorced/widowed, single), region of residence (North, Central, South), area of residence (rural/urban). Weights were used to generate nationally representative results. Dotted lines indicate maximum points for each one of the components. Labeled bars in a dietary component without a common superscript letter differ between education levels, p < 0.05 Bonferroni adjusted. Abbreviations: Veg, vegetables; Whole grains, whole-grain cereals; Leg, legumes; SFE, seafood, poultry, and eggs; Poly fat, polyunsaturated fat; 100% juices, 100% fruit juices; Meat, red and processed meat; Sod, sodium; Sat fat, saturated fat.

**Fig 2:**
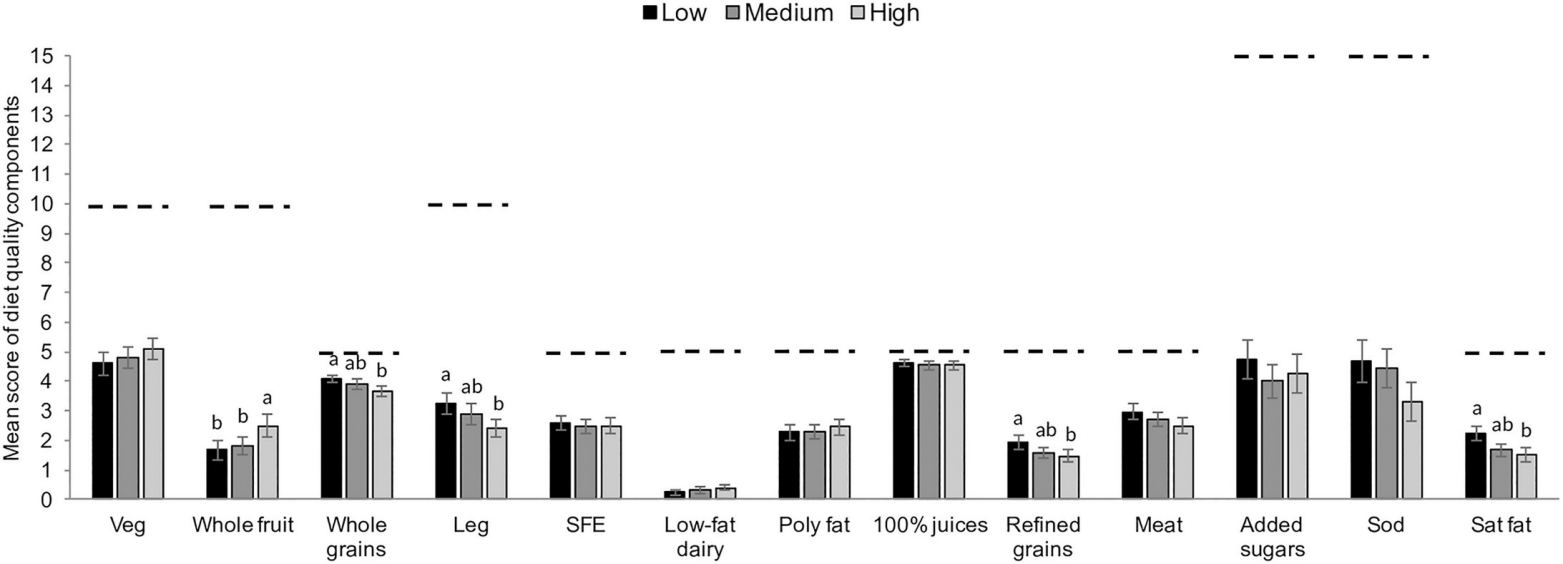
Predicted mean score of diet quality components by tertiles of assets index. Predicted means ± 95% confidence intervals of diet quality components by tertiles of assets index among adults in multivariable-adjusted + educational level models. Predicted values were obtained using linear regression models adjusted for age (continuous), sex, total energy intake, alcohol intake (yes, no), smoking status (current, former, never), employment status (employed, homemaker, other), marital status (married, in union, separated/divorced/widowed, single), region of residence (North, Central, South), area of residence (rural/urban). Weights were used to generate nationally representative results. Dotted lines indicate maximum points for each one of the components. Labeled bars in a dietary component without a common superscript letter differ between education levels, p < 0.05 Bonferroni adjusted. Abbreviations: Veg, vegetables; Whole grains, whole-grain cereals; Leg, legumes; SFE, seafood, poultry, and eggs; Poly fat, polyunsaturated fat; 100% juices, 100% fruit juices; Meat, red and processed meat; Sod, sodium; Sat fat, saturated fat.

In multivariable-adjusted + assets index models, the scores for whole-grains cereals, legumes, and 100% fruit juices components were higher in adults with no reading/writing skills in comparison to adults with ≥ 10 years of school (*p* < 0.05). Likewise, the score for saturated fat component was higher in lower- than higher-educated adults (*p* < 0.05) (Fig 1). Likewise, the scores for whole-grain cereals, legumes, refined grains, and saturated fat components were higher in adults with low versus high assets index, in multivariable-adjusted + educational level models (*p* < 0.05). On the other hand, the score for whole fruit component was higher in adults with high versus low assets index (Fig 2).

### Results of sensitivity analyses

Trends in associations of educational level and tertiles of assets index with total dietary score were similar to those observed when we did not consider corn tortilla as whole-grain cereal. Generally, however, total dietary scores were lower than those observed when corn tortilla was considered as whole-grain cereal (S2 and S3 Tables). Likewise, estimated associations of educational level and tertiles of assets index with total dietary score were similar to those observed when models were further adjusted for BMI categories as well as physical activity and time spent sitting (S2 and S3 Tables). Finally, the trend in the association between educational level and total dietary score was similar to the observed when the category of “reading/writing skills or 3–9 years of school” was divided into two new categories (S4 Table).

## Discussion

The association between SES and diet quality has been barely studied in Mexican adults despite its relevance given that this middle-income country has experienced a rapid nutrition transition, from a traditional to a Western dietary pattern [57]. Furthermore, it is not well understood how educational level and assets index, two different indicators of SES, explain the variability in diet quality among Mexican adults. We observed that the overall diet quality score was higher in lower- than higher-educated Mexican adults. Likewise, the assets index was inversely associated with the total diet quality score. Finally, we found that sex did not modify the association of these two indicators of SES with diet quality.

Systematic reviews based on data from low- to middle-income countries have shown variation in dietary patterns by national income or SES, which would indicate that the SES may play an important role in diet quality variations [58, 59]. Likewise, studies in Brazilian and Chinese individuals, published after the systematic reviews, found inconsistent associations between SES and diet quality [22, 23], which may be due to the use of different indicators of SES as well as different definitions of diet quality. It is important to consider that these studies were conducted in a wide variety of years, probably representing different stages of the nutrition transition. Evidence from high-income countries indicate that the nutrition transition affects individuals with higher SES first, but this may be reversed with the progression of such transition [60–62]. Therefore, our results suggest that Mexican adults are still at an early stage of the nutrition transition. Future research on trends in diet quality by educational level and tertiles of assets index would provide better knowledge about the nutrition transition in Mexico.

Theoretically, a higher level of educational level and assets index may influence the acquisition of healthier lifestyles, including better diet quality, as well as problem-solving capacity and values. Therefore, we expected to find a positive association of educational level and assets index with diet quality among adults. A possibility is that other sociodemographic factors may modify the association of educational level and assets index with diet quality in adults. A previous study showed that, among urban women in the highest tertile of assets index, one level lower of education was associated with higher obesity prevalence, while among the urban women in the lowest tertile of assets index, lower education was associated with lower obesity [63]. We were not able to test whether other sociodemographic factors modified the association of educational level and assets index with diet quality because of sample size constraints. More research will be needed to understand how educational level and the assets index interacts with other sociodemographic factors.

That the overall diet quality was higher in adults with lower educational level or assets index may be explained by the observed higher consumption of whole-grain cereals and legumes in adults with lower than higher SES. Flores et al. 2010 analyzed data from adults who participated in the ENSANUT 2006 and found that individuals with a *traditional* pattern (based primarily on corn and corn-based foods), had lower SES. Although we did not explicitly examine the “traditional” dietary pattern, our results that adults with lower SES had higher intakes of whole-grain cereals and legumes suggests that adults with lower SES still maintain a traditional diet or at a minimum have less resources to use to purchase modern packaged processed foods which tend to be much less healthful. In 2012, the purchase of packaged foods classified as *less-healthy* (e.g., sweets, desserts, and salty snacks) was, on average 55% lower in Mexican urban households with low SES relative to Mexican urban households with high SES [64].

Findings from individual components were either statistically non-significant or consistent with the overall diet quality score. The only exception was the whole fruit component score that was higher in adults with higher than lower assets index. Specifically, we found that the higher consumption of whole-grain cereals and legumes in adults with lower versus higher SES could also indicate a lack of dietary diversity in individuals with lower SES, not only because the whole-grain cereals component was the only one close to the maximum points in adults with the lowest level of education, but also because their scores for vegetables, whole fruit, and total diet quality were less than half of maximum value. Higher diet costs have been associated with lower consumption of vegetables and fruits [65]. Therefore, our results may reflect socioeconomic constraints to eating a varied diet in adults with lower SES. Moreover, the average score for sodium was around 32% of the maximum points in adults with lower SES and the average scores for added sugars were below 33% of the maximum value in adults across educational levels. These results are consistent with those observed by Aburto et al. 2016 [8], who found that the contribution of sugar-sweetened beverages to total energy intake was high across all SES groups, and would suggest that the consumption of ultra-processed foods, often high in sodium or added sugars, was high in Mexican adults in 2012. Given that our dietary index was not developed to identify diversity, future research will be needed for a better understanding of how overall diet quality is associated with dietary diversity and the degree of food processing.

Finally, contrary to what we expected, we found that the association of the educational level and the assets index with diet quality was not different by sex. This suggests that while sex may affect the relationship, sex is not an important moderator of socio-economic disparities in diet quality. However, previous studies in Mexican population showed that the association of diet quality with BMI, waist circumference, and cardiometabolic-related biomarkers varied by educational level and sex [66, 67]. This suggests that sex still plays an important role in the overall relationship between SES, diet quality, and chronic disease, and future research will be needed to monitor potential changes in the relationship between SES and diet quality over time.

Our study has several limitations. First, the cross-sectional did not allow for any temporal causal inference between indicators of SES and dietary scores. Second, we estimated dietary scores based on a single twenty-four-hour recall, which may not represent the long-term dietary habits of the participants [68]. Third, income is another proxy of SES that could better reflect the availability of economic and material resources and directly determines dietary quality by making healthy and nutritious food more affordable and readily accessible [17]. Unfortunately, we were unable to analyze this proxy of SES because only 50% of adults reported their income. Future research will be needed to understand the association of assets index with diet quality adjusting for income. Last, we used a non-conventional definition of educational level, which considered reading/writing skills. Therefore, results observed in our study may not be comparable with findings in other studies where the educational level is usually defined based only on educational attainment. However, we decided to include reading/writing skills given the potential social and economic implications of this variable in Latin America. Moreover, we conducted further analyses with four more conventional categories of educational level, observing similar results. Despite the limitations, this study provides an insight of the potential disparities of diet quality by SES using detailed dietary and sociodemographic information from a representative sample of adults. However, findings should be considered as preliminary.

## Conclusions

Lower educational level and lower assets index were associated with higher diet quality in adults. Our findings also provide insights into the potential food components that may be improved in different sociodemographic groups. Furthermore, our results underscore the importance of policies focused on improving the diet of Mexican adults as a public health priority given that the diet quality seems to be low in this population, regardless the educational level or assets index.

## Supporting information

S1 Table Number of servings recommended by food group in adults. Mexican Dietary Guidelines.
(DOCX)

S2 Table Total diet quality score by educational level in Mexican adults, without considering corn tortilla as whole-grain cereal and further adjusting for body mass index as well as physical activity and time spent sitting. (DOCX)

S3 Table Total diet quality score by tertiles of assets index in Mexican adults, without considering corn tortilla as whole-grain cereal and further adjusting for body mass index as well as physical activity and time spent sitting. (DOCX)

S4 Table Total diet quality score by educational level (four categories) in Mexican adults. (DOCX)
