# Supplementary material for: Association between socioeconomic status and diet quality in Mexican men and women: A cross-sectional study
Source: PLoS One. 2019 Oct 23;14(10):e0224385. doi: 10.1371/journal.pone.0224385 (PMC6808430; doi:10.1371/journal.pone.0224385)
Supplement: S1 Table — Mexican Dietary Guidelines. (DOCX) [file pone.0224385.s001.docx]

**S1 Table.** Number of servings recommended by food group in adults. Mexican

Dietary Guidelines^a^

| **Food groups** | **Servings per 2 000 kcal/day** |
| --- | --- |
| Vegetables^b^ | 3 |
| Fruit^c^ | 3 |
| Cereals^d^ | 8 |
| Legumes | 2 |
| Foods of animal origin (meat products)^e^ | 3.5 |
| Dairy^f^ | 2 |
| Tap water | 750–2 000 mL |
| Sugars^g^ | 2 |
| Fats^h^ | 5 |

| ^a^ Bonvecchio A et al. Guías alimentarias y de actividad física en contexto de sobrepeso y obesidad en población Mexicana. [Dietary and physical activity guidelines in the context of overweight and obesity in the Mexican population.] Mexico, 2015 (8). |
| --- |
| ^b^ Excluding vegetable juices, starchy vegetables such as potato and corn, and salted or pickled vegetables. |
| ^c^ Excluding fruit juices, which are recommended to be consumed in moderation (< 125 mL/day). |
| ^d^ Whole grains shall consist of the intact, ground, cracked, or flaked caryopsis whose principal components, the starchy endosperm, germ, and bran, are present in the same relative proportions as they exist in the intact grain. Some examples of whole grains are oats, amaranth, corn, brown rice, wheat germ, and bran. Corn tortilla is also considered a whole-grain cereal. |
| ^e^ Less than half of servings should be from meat products with high saturated fat or sodium content, including red and processed meats. |
| ^f^ Only low-fat dairy is recommended. |
| ^g^ The consumption of sugars should not exceed 10% of total energy intake. |
| ^h^ Consumption of saturated fat should not exceed 7% of total energy intake, whereas the consumption of polyunsaturated fat should be between 6% and 10% of total energy intake. |
