# Supplementary material for: Association between socioeconomic status and diet quality in Mexican men and women: A cross-sectional study
Source: PLoS One. 2019 Oct 23;14(10):e0224385. doi: 10.1371/journal.pone.0224385 (PMC6808430; doi:10.1371/journal.pone.0224385)
Supplement: S2 Table — (DOCX) [file pone.0224385.s002.docx]

**S2 Table.** Total diet quality score by educational level in Mexican adults, without considering corn tortilla as whole-grain cereal and further adjusting for body mass index as well as physical activity and time spent sitting (*n* = 2,400)^1^.

|  |  | Educational level with literacy | | | | |
| --- | --- | --- | --- | --- | --- | --- |
|  |  | No reading/writing skills |  | Reading/writing skills or 3-9 y of school |  | ≥ 10 y of school |
|  |  | Mean (95% CI) |  | Mean (95% CI) |  | Mean (95% CI) |
|  |  |  |  |  |  |  |
| Without considering tortilla as whole-grain cereal |  |  |  |  |  |  |
| Unadjusted |  | 39.5 (37.0, 41.9)^a^ |  | 33.2 (32.3, 34.2)^b^ |  | 29.5 (28.1, 30.9)^c^ |
| Multivariable-adjusted^2^ |  | 36.5 (34.1, 38.9)^a^ |  | 32.9 (32.0, 33.8)^b^ |  | 31.0 (29.6, 32.4)^b^ |
| Multivariable-adjusted + tertiles of assets  index |  | 35.9 (33.5, 38.3)^a^ |  | 32.9 (31.9, 33.8)^ab^ |  | 31.4 (29.9, 32.8)^b^ |
|  |  |  |  |  |  |  |
| Further adjusting for body mass index^3^ |  |  |  |  |  |  |
| Multivariable-adjusted^2^ |  | 42.2 (39.6, 44.8) ^a^ |  | 38.2 (37.2, 39.2)^b^ |  | 35.4 (33.9, 37.0)^c^ |
| Multivariable-adjusted + tertiles of assets  index |  | 41.5 (38.9, 44.1) ^a^ |  | 38.1 (37.1, 39.1) ^b^ |  | 35.9 (34.4, 37.5) ^b^ |
|  |  |  |  |  |  |  |
| Further adjusting for physical activity and time spent sitting (*n* = 2,221)^4^ |  |  |  |  |  |  |
| Multivariable-adjusted^2^ |  | 42.8 (40.2, 45.3) ^a^ |  | 38.0 (36.9, 39.0) ^b^ |  | 35.9 (34.3, 37.5)^b^ |
| Multivariable-adjusted + tertiles of assets  index |  | 41.7 (39.2, 44.3)^a^ |  | 37.8 (36.8, 38.8) ^b^ |  | 36.6 (35.0, 38.2)^b^ |
|  |  |  |  |  |  |  |

^1^ Linear regression models were used to predict the mean diet quality score according to educational level with literacy categories. Weights were used to generate nationally representative results. Labeled means in a row without a common superscript letter (a,b,c) differ between educational levels, *p* < 0.05, Bonferroni adjusted.

^2^ Adjusted for age (continuous), sex, total energy intake, alcohol intake (yes, no), smoking status (current, former, never), employment status (employed, homemaker, other), marital status (married, in union, separated/divorced/widowed, single), region of residence (North, Central, South), area of residence (rural/urban).

^3^ Categories of body mass index (normal, overweight, obesity

^4^ Physical activity (inactive, moderately active, active), time spent sitting (≥ or < 525 minutes per day).
