# Supplementary material for: Association between socioeconomic status and diet quality in Mexican men and women: A cross-sectional study
Source: PLoS One. 2019 Oct 23;14(10):e0224385. doi: 10.1371/journal.pone.0224385 (PMC6808430; doi:10.1371/journal.pone.0224385)
Supplement: S3 Table — (DOCX) [file pone.0224385.s003.docx]

**S3 Table.** Total diet quality score by tertiles of assets index in Mexican adults, without considering corn tortilla as whole-grain cereal and further adjusting for body mass index as well as physical activity and time spent sitting (*n* = 2,400)^1^.

|  |  | Tertiles of assets index | | | | |
| --- | --- | --- | --- | --- | --- | --- |
|  |  | Low |  | Medium |  | High |
|  |  | Mean (95% CI) |  | Mean (95% CI) |  | Mean (95% CI) |
|  |  |  |  |  |  |  |
| Without considering tortilla as whole-grain cereal |  |  |  |  |  |  |
| Unadjusted |  | 36.6 (35.5, 37.9)^a^ |  | 32.1 (30.8, 33.4)^b^ |  | 30.2 (29.0, 31.4)^b^ |
| Multivariable-adjusted^2^ |  | 34.7 (33.3, 36.1)^a^ |  | 32.2 (31.0, 33.5)^b^ |  | 31.5 (30.3, 32.7)^b^ |
| Multivariable-adjusted + educational  level |  | 34.3 (32.9, 35.7) |  | 32.1 (30.9, 33.4) |  | 31.9 (30.7, 33.1) |
|  |  |  |  |  |  |  |
| Further adjusting for body mass index^3^ |  |  |  |  |  |  |
| Multivariable-adjusted^2^ |  | 40.4 (36.1, 38.8)^a^ |  | 37.5 (36.1, 38.8)^b^ |  | 36.0 (34.7, 37.3)^b^ |
| Multivariable-adjusted + tertiles of assets  index |  | 39.8 (38.3, 41.3)^a^ |  | 37.3 (36.0, 38.7)^b^ |  | 36.5 (35.2, 37.8)^b^ |
|  |  |  |  |  |  |  |
| Further adjusting for physical activity and time spent sitting (*n* = 2,221)^3^ |  |  |  |  |  |  |
| Multivariable-adjusted^2^ |  | 40.8 (39.3, 42.3)^a^ |  | 37.7 (36.3, 39.1)^b^ |  | 35.6 (34.3, 37.0)^b^ |
| Multivariable-adjusted + educational  Level |  | 40.3 (38.8, 41.8)^a^ |  | 37.7 (36.3, 39.0)^b^ |  | 36.0 (34.7, 37.4)^b^ |
|  |  |  |  |  |  |  |

^1^ Linear regression models were used to predict the mean diet quality score according to assets index categories. Weights were used to generate nationally representative results. Labeled means in a row without a common superscript letter (a,b,c) differ between tertiles of assets index, *p* < 0.05, Bonferroni adjusted.

^2^ Adjusted for age (continuous), sex, total energy intake, alcohol intake (yes, no), smoking status (current, former, never), employment status (employed, homemaker, other), marital status (married, in union, separated/divorced/widowed, single), region of residence (North, Central, South), area of residence (rural/urban).

^3^ Categories of body mass index (normal, overweight, obesity

^4^ Physical activity (inactive, moderately active, active), time spent sitting (≥ or < 525 minutes per day)
