# Supplementary material for: Association between socioeconomic status and diet quality in Mexican men and women: A cross-sectional study
Source: PLoS One. 2019 Oct 23;14(10):e0224385. doi: 10.1371/journal.pone.0224385 (PMC6808430; doi:10.1371/journal.pone.0224385)
Supplement: S4 Table — (DOCX) [file pone.0224385.s004.docx]

**S4 Table.** Total diet quality score by educational level (four categories) in Mexican adults (n = 2,400)^1^.

|  |  | Educational level | | | | |  |
| --- | --- | --- | --- | --- | --- | --- | --- |
|  |  | No reading/writing skills  (23.7%) |  | Reading/writing skills or 3-6 y of school  (45.2%) |  | 7-9 y of school (31.2%) | ≥ 10 y of school |
|  |  | Mean (95% CI) |  | Mean (95% CI) |  | Mean (95% CI) | Mean (95% CI) |
|  |  |  |  |  |  |  |  |
| Unadjusted |  | 45.8 (43.1, 48.5)^a^ |  | 39.9 (38.7, 41.2)^b^ |  | 37.1 (35.4, 38.8)^c^ | 33.6 (32.1, 35.1)^d^ |
| Multivariable-adjusted^2^ |  | 42.2 (39.6, 44.8)^a^ |  | 38.1 (36.8, 39.4)^b^ |  | 38.6 (37.0, 40.3)^ab^ | 35.5 (34.0, 37.1)^c^ |
| Multivariable-adjusted + tertiles of assets  index |  | 41.4 (38.8, 44.0)^a^ |  | 37.8 (36.5, 39.1)^b^ |  | 38.7 (37.1, 40.3)^ab^ | 36.1 (34.5, 37.7)^c^ |
|  |  |  |  |  |  |  |  |

^1^ Linear regression models were used to predict the mean diet quality score according to educational level with literacy categories. Weights were used to generate nationally representative results. Labeled means in a row without a common superscript letter (a,b,c) differ between educational levels, *p* < 0.05, Bonferroni adjusted.

^2^ Adjusted for age (continuous), sex, total energy intake, alcohol intake (yes,no), smoking status (current, former, never), employment status (employed, homemaker, other), marital status (married, in union, separated/divorced/widowed, single) region of residence (North, Central, South), area of residence (rural/urban).
